# Prevalence of pediatric and adult optic neuritis in the United States from 2016 to 2023

**DOI:** 10.1038/s41433-025-03683-8

**Published:** 2025-02-26

**Authors:** Nadia J. Abbass, Jacqueline K. Shaia, Priya Shukla, Devon Cohen, David C. Kaelber, Katherine E. Talcott, Rishi P. Singh

**Affiliations:** 1 Case Western Reserve University School of Medicine, Cleveland, OH USA; 2 Center for Ophthalmic Bioinformatics, Cole Eye Institute, Cleveland Clinic, Cleveland, OH USA; 3 Cleveland Clinic Lerner College of Medicine of Case Western Reserve University, Cleveland, OH USA; 4 Cleveland Clinic Cole Eye Institute, Cleveland, OH USA; 5 Departments of Internal Medicine, Pediatrics, and Population and Quantitative Health Sciences, Case Western Reserve University, Cleveland, OH USA; 6 The Center for Clinical Informatics Research and Education, The MetroHealth System, Cleveland, OH USA; 7 Cleveland Clinic Martin Hospitals, Cleveland Clinic Florida, Stuart, FL USA

**Keywords:** Optic nerve diseases, Epidemiology

## Abstract

**Background/Objectives:**

Data on the prevalence of optic neuritis (ON) is limited with reported rates between 5.5 and 115.3 per 100,000. The US data is even more limited with the largest study performed in a single county, finding a prevalence of 115.3. This study aims to fill the gap in US data on ON.

**Methods:**

This (2016–2023) cross-sectional study included patients with ICD-10 codes of retrobulbar neuritis, other ON, unspecified ON, and excluded those with optic papillitis, nutritional optic neuropathy, toxic optic neuropathy. Data was collected through a series of queries in a large platform (TriNetX, LLC) containing EHR data from over 113 million patients.

**Results:**

In 2023, the prevalence of ON was 51.6 per 100,000 people in the overall population. Females had a 1.31 (95% CI, 1.27–1.36) increased odds of disease compared to males. Investigating racial and ethnic breakdown, the highest prevalence was found in the Black population (57.8/100,000) (OR 1.06 (1.01–1.10)), followed by the White (54.7/100,000) (REF) and then Hispanic or Latino populations (45.8/100,000) (OR 0.84 (0.79–0.89)) in 2023. Stratified by age, those ages 45–54 had the highest prevalence (71.3/100,000). The prevalence of ON increased 1.08 (1.05–1.10) times from 2016–2023, with the greatest increase seen in the Hispanic population. Significant increases in prevalence were also seen in the 0–14, 15–24, and 25–34 age groups.

**Discussion:**

Racial, ethnic and sex disparities are apparent in the distribution of ON, with Black individuals and females affected most often, and an increasing prevalence seen in the Hispanic population. Younger subpopulations also demonstrated significant increases, warranting additional investigation.

## Introduction

Optic neuritis (ON) is characterized by inflammation of the optic nerve with a variety of underlying aetiologies. It typically manifests with unilateral, sudden, painful vision loss, decreased colour vision and contrast sensitivity, and a relative afferent pupillary defect [1, 2]. ON is more common in women, comprising 60–75% of cases [3, 4], and predominantly affects those ages 15 to 45 [5]. While often associated with multiple sclerosis (MS), a systemic demyelinating autoimmune disease [6], ON can also occur in association with other disorders such as neuromyelitis optica spectrum disorder, chronic relapsing inflammatory optic neuropathy, stem from toxins, infections, or can be idiopathic [7–9].

Data regarding the incidence and prevalence of ON are limited. Incidence rates vary considerably, ranging from 0.56 to 5.36 per 100,000 [3, 4, 10–27]. While incidence refers to the rate of new cases in a population over time, prevalence includes all existing cases at a specific time, representing the overall burden of disease. Prevalence rates are infrequently reported and are even less consistent, with observed rates spanning from 5.5 to 115.3 per 100,000 individuals [10, 13, 18, 20, 21, 23, 25]. The notable variability in these rates may in part be accounted for by differing methodologies employed in identifying the existing cases of ON. Geographic disparities are evident, with regions further from the equator exhibiting higher incidence rates [20]. Moreover, a decreased risk is observed in South Asian or mixed-race individuals compared to Caucasians, and Asian countries report lower rates than the United States (US) and Europe [6, 20]. Comprehensive, country-wide studies have been performed in South Korea [25, 28] and Columbia [26], facilitated by national health databases, capturing almost their entire populations. Conversely, in the US, epidemiological data have previously been derived from studies completed in a single county or city [4, 21, 22]. The foundational work by Rodriguez et al., carried out in Olmsted County, MN, reported a prevalence of 115.3 per 100,000 persons from 1985 to 1991 [21]. While this study provides valuable insights into optic neuritis in a sub-population of the US, further research into the incidence and prevalence of ON across the US is necessary.

In a country as racially and geographically diverse as the US, localized data cannot adequately capture the national landscape. This study establishes a prevalence rate of ON in the US over the past eight years, as well as a stratification of the prevalence rates by sex, age, race and ethnicity, providing a more accurate and updated landscape of ON in the US.

## Methods

The data used in this study was collected in March 2024 from the TriNetX US Collaborative Network, which provided access to electronic medical records (EMR) (diagnoses, procedures, medications, laboratory values, genomic information) from approximately 113 million patients, making up about 34% of the US population, from 61 healthcare organizations. TriNetX partners with sites including hospitals, primary care centres, and specialty treatment providers to create an updated and linked research network, with data refreshed every two to four weeks. Patient data in this platform comes from electronic health records and then is aggregated, deidentified and searchable by International Classification of Diseases, Ninth and Tenth Revision (ICD9-10) code. Due to the de-identified nature of the data (per the de-identification standard defined in Section §164.514(a) of the HIPAA Privacy Rule), this study is exempt from ethical approval by the Western Institutional Review Board. The de-identification process is attested by a qualified expert as defined in of the HIPAA Privacy Rule.

Patient populations were identified by ICD-10 encounter diagnosis codes. To identify cases of optic neuritis, ICD-10 H46 code was utilized, including the subcodes for retrobulbar neuritis (H46.1), other optic neuritis (H46.8), and unspecified optic neuritis (H46.9). These codes were chosen to most accurately reflect primary optic neuritis not secondary to another cause. The remaining codes under H46 were utilized as exclusion criteria, namely optic papillitis (H 46.0), nutritional optic neuropathy (H46.2), and toxic optic neuropathy (H46.3). The total number of patients with each of the ICD-10 codes utilized in study are reported in Supplementary Table 1. Sub-populations were identified by age, sex, race and ethnicity.

Data were analysed and reported from 2016 until 2023 and followed a calendar year (January 1– December 31). Data were collected through a series of queries, each yielding a number of patients within a specific population of interest. For each query, criteria for age, sex, race and ethnicity, and year were specified, along with the inclusion and exclusion ICD-10 encounter diagnosis codes. Current age at time of data collection (March 2024) was utilized to generate the age groupings. Information regarding race and ethnicity is drawn from the EMR and as such, may either be self-reported or observed by a provider. To calculate prevalence, additional queries were performed to determine the baseline number of patients in the same population. For instance, to determine the prevalence of ON in Hispanic or Latino females ages 15–24 in 2023, the platform was queried to find the total number of patients in this demographic. A query was then conducted to determine the number of patients in this group who met all inclusion and exclusion criteria for an ICD-10 encounter diagnosis of primary ON. The prevalence was calculated by dividing the number of patients with ON by the total number of patients in this population.

### Statistics

Prevalence odds ratios and 95% confidence intervals were calculated and reported. The margin of error and 95% confidence interval for the ratio of two proportions of the 2023/2016 prevalence rates were also calculated and reported. Statistical analysis was carried out using Microsoft excel and R studio. To account for multiple testing, the significant size of the platform and to avoid overinterpretation, calculations with 95% confidence intervals >1.1 or <0.9 were considered to be statistically significant. This study followed the Strengthening the Reporting of Observational Studies in Epidemiology (STROBE) guidelines.

## Results

**Fig. 1:**
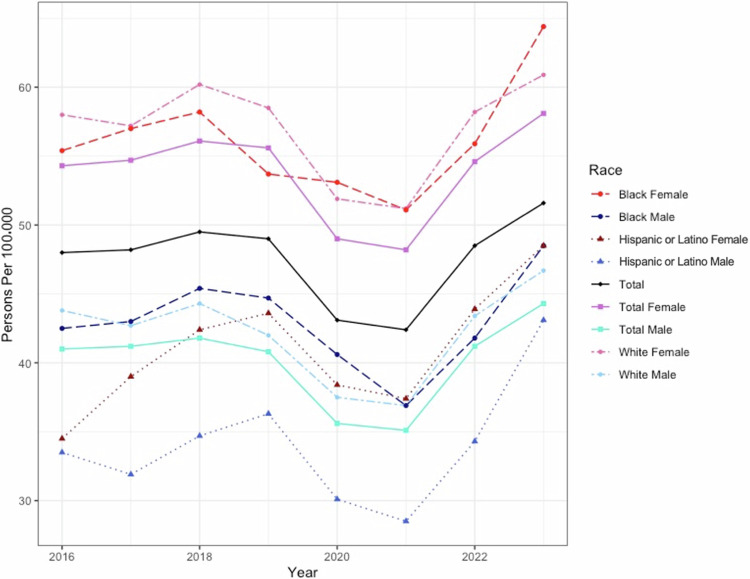
Prevalence of optic neuritis^1^. The yearly total optic neuritis prevalence rate, along with the prevalence rate in populations stratified by sex, ethnicity, and race are shown per 100,000 persons for the duration of the study period (2016–2023).^1^Some populations were too small to yield an accurate number, as the TriNetX system rounds up any single digit. These data points (Black and Hispanic males and females ages 0–14 in 2016 and Hispanic males ages 0–14 in 2017) were included in the calculations of total rates per race/ethnicity group in Fig. 1, understanding that these groups were likely overestimated due to the rounding error. It was decided to include them in analysis of the total population because excluding the 0–14 group entirely would result in falsely elevated rates for the total population.

**Fig. 2:**
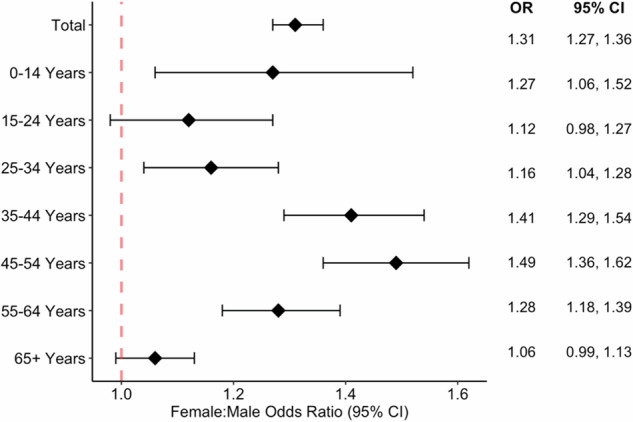
Female to male prevalence odds ratio. The female to male prevalence odds ratio in the total population, as well as the prevalence odds ratios in populations stratified by age, are represented. The line at 1.00 represents the significance threshold. The bars on either side of the center point represent the 95% confidence interval. OR odds ratio, CI confidence interval.

Of 113 million patients represented in the TriNetX US Collaborative Network, 14,870 people had an ICD-10 encounter diagnosis of ON in 2023, consisting of 60.7% females, 35.6% males and the remaining unknown, with a mean age of 52 ± 19 years. The overall prevalence of ON in the US in 2023 was 51.6 per 100,000 people. This represents a non-significant, 1.08 (95% confidence interval (CI) 1.05, 1.10) times increase from the 2016 prevalence, which was 48.0 per 100,000 (Fig. 1). Females were affected more often than males with prevalence rates of 54.3 and 41.0 per 100,000 persons, respectively, and a prevalence odds ratio (POR) of 1.31 (1.27, 1.36). Females were affected at a significantly higher rate in nearly all age groups with the greatest female: male POR in those ages 45–54 (Fig. 2).

When broken down by race and ethnicity, the Black population was most affected with a rate of 57.8, followed by White individuals at 54.7 and Hispanic or Latino individuals at 45.8 per 100,000 persons in 2023. Using the White population as the reference group, the POR for the Black population in 2023 was 1.06 (1.01, 1.10), while the POR for the Hispanic or Latino population was 0.84 (0.79, 0.89).

Stratifying by both race and ethnicity and sex, Black females in 2023 had the highest prevalence at 64.4, followed by White females at 60.8 per 100,000. The lowest prevalence was seen in Hispanic or Latino males at 43.1 per 100,000. Table 1 demonstrates that out of the Black, White, and Hispanic or Latino males and females, Hispanic or Latino females followed by Hispanic or Latino males had the largest increases in prevalence from 2016 to 2023. The prevalence increased 1.41(1.24, 1.58)-fold for Hispanic or Latino females and 1.29 (1.11, 1.46)-fold for Hispanic or Latino males since 2016.

**Table 1:** Change in prevalence of optic neuritis by race and sex from 2016 to 2023.

	2016 prevalence (per 100,000) *(Cases/baseline population)*	2023 prevalence (per 100,000) *(Cases/baseline population)*	Change (2023 prevalence/ 2016 prevalence) (95% CI)
Race, ethnicity and sex			
*White females*	58.0 *(4563/ 7,863,804)*	60.9 *(5980/ 9,821,286)*	1.05 (1.01, 1.09)
*White males*	43.8 *(2668/ 6,088,186)*	46.7 *(3557/ 7,613,580)*	1.07 (1.01, 1.12)
*Black females*	55.4 *(1018/ 1,837,416)*	64.4 *(1608/ 2,498,027)*	1.16 (1.07, 1.25)
*Black males*	42.5 *(557/ 1,311,459)*	48.5 *(862/ 1,776,051)*	1.14 (1.02, 1.26)
*Hispanic or Latino females*	34.4 *(425/ 1,236,420)*	48.5 *(819/ 1,689,781)*	1.41 (1.24, 1.58)
*Hispanic or Latino males*	33.5 *(314/ 936,488)*	43.1 *(554/ 1,284,245)*	1.29 (1.11, 1.46)
Age group			
*Total*	48.0 *(10,640/ 22,182,938)*	51.6 *(14,870/ 28,831,992)*	1.08 (1.05, 1.10)
*0*–*14*	3.4 *(76/ 2,235,291)*	10.1 *(484/ 4,806,253)*	2.96 (2.25, 3.68)
*15*–*24*	20.1 *(498/ 2,471,859)*	30.7 *(967/ 3,146,441)*	1.53 (1.36, 1.69)
*25*–*34*	35.2 *(856/ 2,432,094)*	53.3 *(1675/ 3,144,449)*	1.51 (1.39, 1.64)
*35*–*44*	59.7 *(1502/ 2,514,733)*	70.0 *(2349/ 3,354,417)*	1.17 (1.10, 1.25)
*45*–*54*	72.3 *(1765/ 2,442,565)*	71.3 *(2364/ 3,315,625)*	0.99 (0.93, 1.05)
*55*–*64*	65.3 *(1996/3,058,580)*	65.9 *(2581/ 3,914,053)*	1.01 (0.95, 1.07)
*65*+	56.2 *(3947/ 7,027,816)*	62.2 *(4450/ 7,150,754)*	1.11 (1.06, 1.16)

**Fig. 3:**
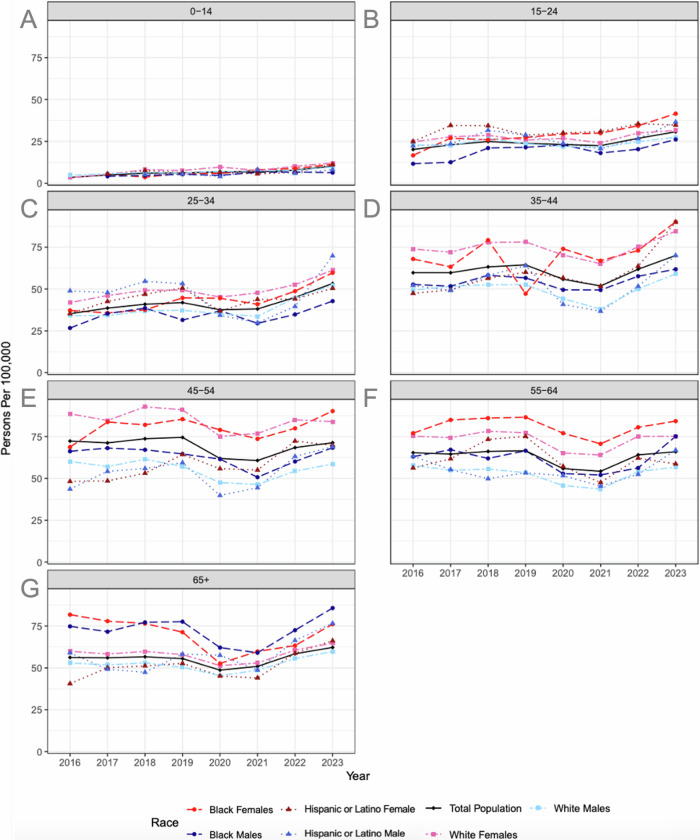
Stratified prevalence of optic neuritis^1^. The total prevalence of optic neuritis in each age group (**A**–**G**), as well as the prevalence stratified by race, ethnicity, and sex, are shown per 100,000 persons for the duration of the study period (2016–2023). ^1^Some populations were too small to yield an accurate number, as the TriNetX system rounds any single digit. These data points (Black and Hispanic males and females ages 0–14 in 2016 and Hispanic males ages 0–14 in 2017) were excluded from Fig. 3 and from analysis of trends in these populations, with data points and analysis starting the year reliable data was able to be extracted.

The youngest population, those ages 0–14, had the lowest prevalence with a rate of 10.1 per 100,000 in 2023, making up just 3% of ON cases that year. Since 2016, this population had a 2.96 (2.25, 3.68) times increase, the largest increase of any age group (Table 1). When stratified by sex, race and ethnicity, White females had the greatest increase of any subgroup, with a prevalence of 3.3 in 2016 and 12.0 in 2023, a 3.65 (1.86, 5.44)-fold increase. The populations of Black and Hispanic or Latino males and females in 2016 and Hispanic or Latino males in 2017 were too small to reliably count in the platform, as it rounds any result between 1 and 10 to 10 for statistical de-identification (Fig. 3A).

For the population ages 15–24, the ON prevalence was 30.7 per 100,000 in 2023, an increase of 1.53 (1.36, 1.69) times since 2016. Further breakdown of this increase demonstrates that it is most significant in the Black population with the 2023 rate being 2.50 (1.56, 3.45) times greater in Black females and 2.25 (1.18, 3.33) times greater in Black males than their respective rates in 2016 (Fig. 3B).

The 25–34 age group demonstrated a prevalence of 53.3 per 100,000 in 2023. This population experienced a 1.51(1.39, 1.64) times increase from 2016 to 2023. In this age group, all races/ethnicities had comparable increases in prevalence from 2016 to 2023 (Fig. 3C). Despite having the lowest prevalence when accounting for all age groups, Hispanic or Latino males have the highest rate in the 25–34 age group, with a prevalence of 69.8 per 100,000 in 2023.

Those 35–44 years old had the second highest prevalence of all age groups, 70.0 per 100,000 in 2023, an increase by 1.17 (1.10, 1.25) times since 2016. Within this population, Hispanic or Latino females had the greatest increase of 1.90 (1.41, 2.38) times (Fig. 3D).

The highest prevalence was found in those ages 45–54 with a rate of 71.3 per 100,000 in 2023. This population remained constant between 2016 and 2023, with the 2023 prevalence rate being 99% of the 2016 prevalence rate (Fig. 3E). This group had the largest sex differential with a female : male POR of 1.49 (1.36, 1.62) (Fig. 2).

The prevalence in the 55–64 group was 65.9 per 100,000 in 2023. This population also remained constant with a 1.01 (0.95, 1.08) times increase since 2016. When stratified by sex, race and ethnicity, all sub populations were similarly constant throughout this time period (Fig. 3F).

In the oldest age group, those 65 and over, the prevalence was found to be 62.2 per 100,000 in 2023. This population had a modest, non-significant increase of 1.11 (1.06, 1.16) times since 2016. Although males generally had a lower prevalence rate of ON, Black males predominated in this age group, with a prevalence of 85.7 per 100,000 in 2023 (Fig. 3G).

The total number of cases and baseline patients for each year stratified by age, sex, race, and ethnicity are reported in Supplementary Tables 2–9.

## Discussion

As confirmed by our literature review, there is a paucity of data on the prevalence of ON in the US, particularly concerning trends over time and subgroup disparities. To address this, we analysed data from EMR records of over 113 million patients. Our findings indicate a prevalence of ON in the US of 51.6 per 100,000 people in 2023. Females were predominantly affected with a female: male POR of 1.31. The highest prevalence was found in the Black population, with a similar rate in the White population, and the lowest prevalence seen in the Hispanic or Latino population. Stratifying by age, those ages 45–54 had the highest prevalence, while the 0–14 group demonstrated the lowest prevalence. From 2016 to 2023, the 0–14 age group had the largest prevalence increase, followed by those 15–24. Overall, there is a trend of greater increases in prevalence in the younger age groups compared to the older age groups, many of which remained relatively constant (Table 1).

Our prevalence rate is substantially lower than the previously reported rate of 115.3 per 100,000 in the study by Rodriguez et al., performed in Olmsted County, Minnesota [21]. ON is often associated with MS, a condition known to occur more often at higher latitudes, which may explain the disparity between these results [29]. Additionally, the study by Rodriguez et al. was based on data from the Mayo Clinic system, where the study population may be more likely than the general population to have access to specialized care to receive this diagnosis. The only other study reporting prevalence in the US took place in Hawaii, with a rate of 5.5 per 100,000 [13]. Other studies taking place outside the US have reported rates ranging from 7.87 for the prevalence of acute ON in Barcelona [23] to 114.8 for the ON prevalence in the United Kingdom [20].

Our study found that ON more predominantly affects females where females have a 31% increased odds of ON compared to males, a finding in line with many other studies [15, 16, 18, 20, 21, 24–26]. One likely explanation for this finding is a greater prevalence of demyelinating diseases like MS in females [30]. Another proposed mechanism is the presence of multiple immune regulation genes on the X chromosome, increasing the risk of all autoimmune phenomena in females [20, 31]. Lee et al. found a female: male prevalence ratio of 1.14 in adults with ON in South Korea, which ranged from 1.00 to 1.56 in different age groups [25]. Braithwaite et al. similarly describes a female predominance with an adjusted incidence rate ratio of 2.26 [20].

Concerning the racial and ethnic distribution of ON, our study found a similar rate in the Black population (POR 1.06) and a significantly lower rate in the Hispanic or Latino population (POR 0.84) compared the White population in 2023. This finding has not previously been reported in the literature. Although the Hispanic or Latino population had the lowest rate of ON, the prevalence in this group appears to be growing, with a 1.41-fold increase in Hispanic or Latino females and 1.29-fold increase in Hispanic or Latino males since 2016. A study performed in Hawaii found that those of Japanese, Chinese, and Filipino descent had approximately half the prevalence rate of ON compared to Whites. This study was very limited, however, as it was based on only 36 cases of ON and was performed in only a single state whose racial breakdown does not reflect that of the general US [13]. The other available study reporting the prevalence of ON in the US, performed in Olmsted County, Minnesota, did not report a breakdown by race or ethnicity [21]. Although MS is one of the conditions commonly related to ON, the racial breakdown of MS in the US does not mirror our findings for ON, as MS has a significant predominance in the White population, compared to the Black and Hispanic or Latino populations [32]. It is important to note, however, that conversion rates to MS after a diagnosis of ON range from 8.3 to 40% [33, 34], meaning that a majority of patients with ON do not develop MS. This highlights the importance of examining ON as its own entity, related to but still separate from MS and other demyelinating diseases.

Examining the prevalence rate by age group, our findings revealed peak prevalence in those ages 45–54 years old, closely followed by those 35–44 years old. These findings are in line with other studies reporting peak prevalences in age groups 40–59 [24], 45–49 [26], and 50–54 [25]. On the other hand, the lowest rate of ON was found in the youngest population, those ages 0–14. Although this is the smallest population, it had the most significant increase in prevalence, almost tripling from the 2016 rate of 3.4 per 100,000. A recent study of ON in South Korea also reported an increasing prevalence in the paediatric population. This could be a reflection of trends toward younger puberty, especially since they reported peak paediatric prevalence in the 10–14 age range [25]. Prevalence of paediatric ON is rarely reported and no other study has reported trends in the prevalence rates over time. Further investigation of paediatric ON is warranted to understand what is driving this phenomenon. Additionally, greater awareness of this trend is needed so that paediatric patients experiencing signs and symptoms of ON can be evaluated and diagnosed more readily.

Finally, it should be noted that a period shift can be seen in Figs. 1 and 3 with a dip in prevalence occurring in 2020 likely secondary to the COVID-19 pandemic. The underutilization of healthcare during this time likely resulted in an underestimation of the ON prevalence in 2020 and following years until healthcare utilization returned to pre-pandemic levels. Additionally, multiple case reports have postulated a connection between ON and both COVID-19 infection [35] and vaccination [36], with another study demonstrating a significantly increased risk of ON after COVID-19 infection, compared to COVID-19 vaccination [37]. Despite the increased risk associated with COVID-19 infection, ON rates in 2020 and 2021 remain lower than pre-pandemic levels, further demonstrating the effect of healthcare underutilization and emphasizing the probable underestimation during these years.

### Limitations

This study has several limitations. Although broadly, the population of patients in TriNetX closely resembles the general population of the US, there are still some meaningful differences in representation. White and Black patients represent 54% and 13% of the platform respectively, closely reflecting the US Census data. Hispanic or Latino patients, however, are somewhat underrepresented, making up 8.8% of patients in the platform but 19.1% of the general population according to the US census in 2022. Underrepresentation of Hispanic or Latino patients could lead to less accurate conclusions about this population compared to other groups. Additionally, since the Hispanic or Latino population demonstrated the lowest prevalence rate, underrepresentation of this population may result in an overestimation of the total US prevalence rate. Furthermore, since information regarding race and ethnicity in the EMR can either be self-reported or recorded by a provider, there may be errors in recording race if a patient selects a race different than what they identify as or if a provider incorrectly assumes a patient’s race.

TriNetX also includes data from patients in all regions of the United States, however, there are also some discrepancies in geographic representation. The Northeast region is overrepresented, making up 29% of patients in the database, compared to 17% of the population in the latest US census. The West and Midwest are slightly underrepresented, making up 13 and 15% of the database, but 24 and 21% of the US census, respectively. The South is proportionally represented as 38% of the database and 39% of the US census. Over-representation of more northern populations could lead to an overestimation of the prevalence of ON, as the prevalence of demyelinating diseases increases farther from the equator.

Furthermore, some small sub-population counts were rounded to 10 due to the platform’s deidentification requirements, resulting in the rounding of all counts 1–9 up to 10. This rounding affected a few populations, namely Black and Hispanic or Latino males and females ages 0–14 in 2016 and Hispanic or Latino males ages 0–14 in 2017, limiting our ability to draw fully accurate conclusions about these sub-populations. The reported rates are likely an overestimation, with the true number of cases in these populations being less than 10. This study is also constrained by reliance on ICD-10 diagnosis codes, which may not always be accurately applied in clinical settings. We cannot verify that the diagnosis was correctly assigned given lack of chart access. Furthermore, this method excludes those with signs/symptoms of ON who were not able to be seen and diagnosed by a specialist, contributing to a potential underestimation of the prevalence.

### Conclusion

Available data on the prevalence of ON in the United States is sparse, outdated, and not generalizable to the entire US population, underpinning the need for our analysis. Our findings revealed a prevalence of 51.6 cases of ON per 100,000 people in 2023 in the general population. This population consisted of 60.7% females where females had a 31% increased odds of disease compared to males. Investigating the racial and ethnic breakdown, we found the highest prevalence in the Black population (57.8), followed by the white (54.7) and then Hispanic or Latino populations (45.8) in 2023. When stratified by age group, those ages 45–54 had the highest prevalence rate of ON. Substantial increases in prevalence rates were seen in the 0–14, 15–24, and 25–34 age groups with 2.96-, 1.52- and 1.51-fold increases, respectively. Additional research is needed to determine the driving force behind the increasing rates of ON in these younger populations.

## Summary

### What was known before

- Data on the prevalence of optic neuritis (ON) is limited with reported rates between 5.5 and 115.3 per 100,000.
- The latest and only large prevalence study carried out in the United States encompassed data from 1985 to 1991 and did not include information on race or ethnicity.
- Optic neuritis has been shown to be associated with COVID-19 infection but no US prevalence study has examined the rate of optic neuritis since the pandemic.

### What this study adds

- Optic neuritis most often affects female and Black individuals.
- The prevalence of optic neuritis has remained stable in the general population from 2016 to 2023.
- The Hispanic or Latino populations and younger populations have demonstrated significant increases in the prevalence of optic neuritis.

## Supplementary information

Supplemental Tables

## Data Availability

All data collected in this study have been shared in the supplemental files.
