# Supplementary material for: Prevalence of pediatric and adult optic neuritis in the United States from 2016 to 2023
Source: Eye (Lond). 2025 Feb 26;39(8):1608–14. doi: 10.1038/s41433-025-03683-8 (PMC12089471; doi:10.1038/s41433-025-03683-8)
Supplement: Supplementary file 1 — Supplemental Tables [file 41433_2025_3683_MOESM1_ESM.docx]

**Supplemental Table 1.** Total Number of Patients with Each Inclusion and Exclusion ICD-10 Diagnosis Code

| ICD-10 Code | Diagnosis | Total Number of Cases * |
| --- | --- | --- |
| H46.1 | Retrobulbar Neuritis | 5,187 |
| H46.8 | Other Optic Neuritis | 41,562 |
| H46.9 | Unspecified Optic Neuritis | 89,877 |
| H46.0 | Optic Papillitis | 5,583 |
| H46.2 | Nutritional Optic Neuropathy | 1,154 |
| H46.3 | Toxic Optic Neuropathy | 1,363 |

*This data was collected on January 1, 2025, after the original data collection occurred. Since TriNetX is a live platform that is constantly updated, these values are likely slightly different than the corresponding values when the main data collection occurred.

**Supplemental Table 2.** Optic Neuritis Cases and Baseline Populations in 2016

|  | All Sexes | | Females | | Males | |
| --- | --- | --- | --- | --- | --- | --- |
| Age | Optic Neuritis Cases | Baseline Population | Optic Neuritis Cases | Baseline Population | Optic Neuritis Cases | Baseline Population |
| All Races and Ethnicities | | | | | | |
| 0-14 | 76 | 2,235,291 | 33 | 1,026,445 | 43 | 1,171,344 |
| 15-24 | 498 | 2,471,859 | 274 | 1,202,454 | 223 | 1,214,596 |
| 25-34 | 856 | 2,432,094 | 567 | 1,455,279 | 276 | 899,259 |
| 35-44 | 1,502 | 2,514,733 | 1,020 | 1,540,534 | 443 | 889,238 |
| 45-54 | 1,765 | 2,442,565 | 1,164 | 1,423,737 | 545 | 930,291 |
| 55-64 | 1,996 | 3,058,580 | 1,239 | 1,693,531 | 705 | 1,242,011 |
| 65+ | 3,947 | 7,027,816 | 2,262 | 3,748,065 | 1,579 | 2,946,747 |
| Total | 10640 | 22,182,938 | 6559 | 12,090,045 | 3814 | 9,293,486 |
| White Population | | | | | | |
| 0-14 | 48 | 1,178,721 | 18 | 547,795 | 30 | 630,606 |
| 15-24 | 335 | 1,409,207 | 173 | 702,644 | 162 | 705,975 |
| 25-34 | 551 | 1,417,216 | 365 | 870,833 | 186 | 546,005 |
| 35-44 | 969 | 1,495,445 | 697 | 943,643 | 272 | 551,358 |
| 45-54 | 1,163 | 1,503,965 | 803 | 906,604 | 359 | 596,976 |
| 55-64 | 1,350 | 1,991,705 | 862 | 1,146,623 | 488 | 844,591 |
| 65+ | 2,816 | 4,960,057 | 1,645 | 2,745,662 | 1,171 | 2,212,675 |
| Total | 7232 | 13,956,316 | 4563 | 7,863,804 | 2668 | 6,088,186 |
| Black Population | | | | | | |
| 0-14 | 11 | 414,293 | 10 | 195,486 | 10 | 218,712 |
| 15-24 | 58 | 412,816 | 34 | 205,250 | 24 | 207,311 |
| 25-34 | 143 | 428,149 | 102 | 274,146 | 41 | 153,806 |
| 35-44 | 255 | 407,996 | 179 | 263,777 | 76 | 144,095 |
| 45-54 | 250 | 369,035 | 157 | 228,432 | 93 | 140,494 |
| 55-64 | 312 | 438,242 | 198 | 257,103 | 114 | 181,032 |
| 65+ | 537 | 679,487 | 338 | 413,222 | 199 | 266,009 |
| Total | 1566 | 3,150,018 | 1018 | 1,837,416 | 557 | 1,311,459 |
| Hispanic or Latino Population | | | | | | |
| 0-14 | 11 | 453,435 | 10 | 212,551 | 10 | 240,827 |
| 15-24 | 103 | 437,241 | 54 | 217,050 | 49 | 220,153 |
| 25-34 | 111 | 277,594 | 64 | 181,431 | 47 | 96,127 |
| 35-44 | 128 | 261,525 | 83 | 175,169 | 45 | 86,327 |
| 45-54 | 112 | 240,497 | 74 | 153,416 | 38 | 87,064 |
| 55-64 | 123 | 208,386 | 70 | 124,537 | 53 | 83,824 |
| 65+ | 142 | 294,481 | 70 | 172,266 | 72 | 122,166 |
| Total | 730 | 2,173,159 | 425 | 1,236,420 | 314 | 936,488 |

**Supplemental Table 3.** Optic Neuritis Cases and Baseline Populations in 2017

|  | All Sexes | | Females | | Males | |
| --- | --- | --- | --- | --- | --- | --- |
| Age | Optic Neuritis Cases | Baseline Population | Optic Neuritis Cases | Baseline Population | Optic Neuritis Cases | Baseline Population |
| All Races and Ethnicities | | | | | | |
| 0-14 | 139 | 2,835,419 | 66 | 1,304,408 | 73 | 1,485,579 |
| 15-24 | 662 | 2,885,122 | 388 | 1,424,688 | 273 | 1,396,686 |
| 25-34 | 1,122 | 2,903,833 | 738 | 1,757,039 | 359 | 1,061,655 |
| 35-44 | 1,800 | 3,014,347 | 1,212 | 1,834,702 | 546 | 1,087,433 |
| 45-54 | 2,071 | 2,910,165 | 1,369 | 1,689,973 | 643 | 1,123,575 |
| 55-64 | 2,346 | 3,635,227 | 1,469 | 2,007,650 | 829 | 1,493,681 |
| 65+ | 4,560 | 8,145,464 | 2,625 | 4,364,478 | 1,837 | 3,429,947 |
| Total | 12700 | 26329577 | 7867 | 14382938 | 4560 | 11078556 |
| White Population | | | | | | |
| 0-14 | 82 | 1,515,956 | 37 | 706,180 | 45 | 809,239 |
| 15-24 | 418 | 1,660,389 | 233 | 841,077 | 184 | 818,553 |
| 25-34 | 710 | 1,710,365 | 489 | 1,061,372 | 221 | 648,383 |
| 35-44 | 1,165 | 1,809,879 | 814 | 1,131,306 | 350 | 677,893 |
| 45-54 | 1,328 | 1,805,847 | 915 | 1,081,558 | 413 | 723,687 |
| 55-64 | 1,576 | 2,390,742 | 1,016 | 1,368,086 | 560 | 1,021,766 |
| 65+ | 3,205 | 5,790,776 | 1,869 | 3,207,409 | 1,336 | 2,580,385 |
| Total | 8484 | 16683954 | 5373 | 9396988 | 3109 | 7279906 |
| Black Population | | | | | | |
| 0-14 | 24 | 511,798 | 13 | 241,255 | 11 | 270,377 |
| 15-24 | 93 | 470,866 | 64 | 238,086 | 29 | 232,477 |
| 25-34 | 175 | 492,200 | 113 | 316,303 | 62 | 175,663 |
| 35-44 | 276 | 467,611 | 189 | 298,980 | 87 | 168,452 |
| 45-54 | 327 | 421,271 | 216 | 258,156 | 111 | 162,954 |
| 55-64 | 385 | 497,107 | 246 | 289,851 | 139 | 207,078 |
| 65+ | 568 | 753,716 | 356 | 457,240 | 212 | 296,061 |
| Total | 1848 | 3614569 | 1197 | 2099871 | 651 | 1513062 |
| Hispanic or Latino Population | | | | | | |
| 0-14 | 22 | 537,300 | 14 | 252,320 | 10 | 284,882 |
| 15-24 | 137 | 471,388 | 82 | 237,512 | 55 | 233,793 |
| 25-34 | 135 | 304,413 | 86 | 202,108 | 49 | 102,226 |
| 35-44 | 144 | 291,769 | 96 | 194,025 | 48 | 97,694 |
| 45-54 | 135 | 267,114 | 82 | 169,209 | 53 | 97,871 |
| 55-64 | 137 | 231,832 | 85 | 137,680 | 52 | 94,121 |
| 65+ | 160 | 321,814 | 94 | 187,704 | 66 | 134,050 |
| Total | 870 | 2425630 | 539 | 1380558 | 333 | 1044637 |

**Supplemental Table 4.** Optic Neuritis Cases and Baseline Populations in 2018

|  | All Sexes | | Females | | Males | |
| --- | --- | --- | --- | --- | --- | --- |
| Age | Optic Neuritis Cases | Baseline Population | Optic Neuritis Cases | Baseline Population | Optic Neuritis Cases | Baseline Population |
| All Races and Ethnicities | | | | | | |
| 0-14 | 193 | 3,237,727 | 94 | 1,488,726 | 99 | 1,692,361 |
| 15-24 | 761 | 3,062,204 | 417 | 1,543,181 | 339 | 1,439,380 |
| 25-34 | 1,252 | 3,062,713 | 826 | 1,860,791 | 384 | 1,098,734 |
| 35-44 | 2,025 | 3,203,496 | 1,375 | 1,924,367 | 593 | 1,170,003 |
| 45-54 | 2,282 | 3,097,736 | 1,496 | 1,782,712 | 704 | 1,201,694 |
| 55-64 | 2,546 | 3,851,216 | 1,605 | 2,108,559 | 865 | 1,587,672 |
| 65+ | 4,718 | 8,334,005 | 2,682 | 4,441,688 | 1,899 | 3,503,572 |
| Total | 13777 | 27849097 | 8495 | 15150024 | 4883 | 11693416 |
| White Population | | | | | | |
| 0-14 | 125 | 1,721,948 | 65 | 802,251 | 60 | 918,552 |
| 15-24 | 486 | 1,751,746 | 260 | 907,926 | 225 | 842,268 |
| 25-34 | 792 | 1,775,704 | 546 | 1,109,993 | 245 | 663,785 |
| 35-44 | 1,300 | 1,907,847 | 918 | 1,179,601 | 381 | 726,144 |
| 45-54 | 1,526 | 1,907,269 | 1,053 | 1,134,775 | 473 | 770,612 |
| 55-64 | 1,717 | 2,510,777 | 1,118 | 1,428,967 | 599 | 1,079,267 |
| 65+ | 3,332 | 5,879,167 | 1,941 | 3,246,930 | 1,391 | 2,623,702 |
| Total | 9278 | 17454458 | 5901 | 9810443 | 3374 | 7624330 |
| Black Population | | | | | | |
| 0-14 | 24 | 582,053 | 10 | 275,215 | 14 | 306,419 |
| 15-24 | 118 | 496,815 | 67 | 258,400 | 50 | 237,913 |
| 25-34 | 198 | 519,411 | 126 | 334,820 | 71 | 184,057 |
| 35-44 | 355 | 497,054 | 249 | 314,353 | 106 | 182,264 |
| 45-54 | 342 | 449,629 | 224 | 273,286 | 118 | 175,984 |
| 55-64 | 398 | 525,064 | 261 | 303,378 | 137 | 221,286 |
| 65+ | 595 | 775,813 | 359 | 469,187 | 236 | 305,674 |
| Total | 2030 | 3845839 | 1296 | 2228639 | 732 | 1613597 |
| Hispanic or Latino Population | | | | | | |
| 0-14 | 37 | 595,560 | 21 | 279,587 | 16 | 315,790 |
| 15-24 | 157 | 476,452 | 84 | 245,032 | 73 | 231,271 |
| 25-34 | 157 | 317,907 | 99 | 211,277 | 58 | 106,473 |
| 35-44 | 174 | 305,404 | 112 | 198,580 | 62 | 106,711 |
| 45-54 | 152 | 280,643 | 93 | 175,056 | 59 | 105,499 |
| 55-64 | 155 | 243,790 | 105 | 143,313 | 50 | 100,376 |
| 65+ | 165 | 332,584 | 99 | 193,316 | 66 | 139,145 |
| Total | 997 | 2552340 | 613 | 1446161 | 384 | 1105265 |

**Supplemental Table 5.** Optic Neuritis Cases and Baseline Populations in 2019

|  | All Sexes | | Females | | Males | |
| --- | --- | --- | --- | --- | --- | --- |
| Age | Optic Neuritis Cases | Baseline Population | Optic Neuritis Cases | Baseline Population | Optic Neuritis Cases | Baseline Population |
| All Races and Ethnicities | | | | | | |
| 0-14 | 220 | 3,626,668 | 116 | 1,671,919 | 104 | 1,891,400 |
| 15-24 | 761 | 3,201,760 | 429 | 1,644,662 | 328 | 1,470,214 |
| 25-34 | 1,347 | 3,216,183 | 892 | 1,959,739 | 415 | 1,142,943 |
| 35-44 | 2,170 | 3,370,722 | 1,457 | 2,008,721 | 638 | 1,243,659 |
| 45-54 | 2,433 | 3,266,796 | 1,593 | 1,869,778 | 739 | 1,275,385 |
| 55-64 | 2,682 | 4,033,654 | 1,672 | 2,197,275 | 914 | 1,672,338 |
| 65+ | 4,695 | 8,460,891 | 2,659 | 4,508,200 | 1,865 | 3,561,387 |
| Total | 14308 | 29176674 | 8818 | 15860294 | 5003 | 12257326 |
| White Population | | | | | | |
| 0-14 | 131 | 1,937,880 | 69 | 906,149 | 62 | 1,030,561 |
| 15-24 | 454 | 1,835,739 | 249 | 969,985 | 204 | 864,107 |
| 25-34 | 830 | 1,849,869 | 574 | 1,161,460 | 256 | 686,394 |
| 35-44 | 1,364 | 2,001,399 | 958 | 1,227,237 | 406 | 771,910 |
| 45-54 | 1,547 | 2,004,048 | 1,079 | 1,185,713 | 467 | 816,280 |
| 55-64 | 1,751 | 2,621,253 | 1,144 | 1,481,991 | 607 | 1,136,530 |
| 65+ | 3,239 | 5,944,295 | 1,899 | 3,278,020 | 1,340 | 2,657,364 |
| Total | 9316 | 18194483 | 5972 | 10210555 | 3342 | 7963146 |
| Black Population | | | | | | |
| 0-14 | 36 | 649,577 | 18 | 306,245 | 18 | 340,070 |
| 15-24 | 128 | 522,087 | 76 | 278,079 | 52 | 243,469 |
| 25-34 | 220 | 550,026 | 159 | 355,793 | 61 | 193,728 |
| 35-44 | 359 | 527,260 | 249 | 527,260 | 110 | 194,311 |
| 45-54 | 372 | 482,290 | 249 | 291,535 | 123 | 190,403 |
| 55-64 | 437 | 560,091 | 280 | 323,601 | 157 | 236,103 |
| 65+ | 597 | 809,822 | 350 | 490,726 | 247 | 318,120 |
| Total | 2149 | 4101153 | 1381 | 2573239 | 768 | 1716204 |
| Hispanic or Latino Population | | | | | | |
| 0-14 | 38 | 655,419 | 19 | 307,910 | 19 | 347,328 |
| 15-24 | 135 | 477,643 | 72 | 250,584 | 63 | 226,894 |
| 25-34 | 176 | 341,994 | 115 | 227,118 | 61 | 114,720 |
| 35-44 | 199 | 324,189 | 125 | 208,245 | 74 | 115,830 |
| 45-54 | 185 | 296,540 | 118 | 183,654 | 67 | 112,781 |
| 55-64 | 169 | 256,342 | 112 | 149,276 | 57 | 106,964 |
| 65+ | 187 | 340,265 | 104 | 197,507 | 83 | 142,619 |
| Total | 1089 | 2692392 | 665 | 1524294 | 424 | 1167136 |

**Supplemental Table 6.** Optic Neuritis Cases and Baseline Populations in 2020

|  | All Sexes | | Females | | Males | |
| --- | --- | --- | --- | --- | --- | --- |
| Age | Optic Neuritis Cases | Baseline Population | Optic Neuritis Cases | Baseline Population | Optic Neuritis Cases | Baseline Population |
| All Races and Ethnicities | | | | | | |
| 0-14 | 237 | 3,497,939 | 129 | 1,618,856 | 107 | 1,818,176 |
| 15-24 | 731 | 3,169,773 | 433 | 1,679,219 | 290 | 1,406,202 |
| 25-34 | 1,330 | 3,537,065 | 885 | 2,133,193 | 409 | 1,289,790 |
| 35-44 | 2,032 | 3,639,412 | 1,398 | 2,134,265 | 573 | 1,386,371 |
| 45-54 | 2,152 | 3,474,263 | 1410 | 1,962,566 | 659 | 1,390,347 |
| 55-64 | 2,332 | 4,179,699 | 1,446 | 2,255,132 | 799 | 1,764,298 |
| 65+ | 4,045 | 8,312,864 | 2,231 | 4,406,641 | 1,642 | 3,541,593 |
| Total | 12859 | 29811015 | 7932 | 16189872 | 4479 | 12596777 |
| White Population | | | | | | |
| 0-14 | 154 | 1,878,623 | 85 | 881,159 | 69 | 996,297 |
| 15-24 | 443 | 1,802,809 | 262 | 979,184 | 180 | 821,438 |
| 25-34 | 820 | 1,802,809 | 557 | 1,233,262 | 262 | 744,688 |
| 35-44 | 1,266 | 2,112,099 | 897 | 1,276,809 | 368 | 832,988 |
| 45-54 | 1,326 | 2,084,146 | 914 | 1,217,248 | 411 | 864,836 |
| 55-64 | 1,514 | 2,672,635 | 976 | 1,495,829 | 537 | 1,173,910 |
| 65+ | 2,811 | 5,778,410 | 1,627 | 3,164,512 | 1,184 | 2,605,539 |
| Total | 8334 | 18131531 | 5318 | 10248003 | 3011 | 8039696 |
| Black Population | | | | | | |
| 0-14 | 31 | 585,735 | 16 | 277,617 | 15 | 319,024 |
| 15-24 | 132 | 499,216 | 81 | 275,681 | 51 | 222,964 |
| 25-34 | 242 | 577,216 | 164 | 368,648 | 77 | 208,026 |
| 35-44 | 359 | 554,737 | 256 | 346,121 | 103 | 208,146 |
| 45-54 | 366 | 508,609 | 241 | 305,083 | 125 | 203,113 |
| 55-64 | 388 | 582,455 | 257 | 333,818 | 131 | 248,217 |
| 65+ | 464 | 824,317 | 261 | 496,330 | 203 | 327,019 |
| Total | 1982 | 4132285 | 1276 | 2403298 | 705 | 1736509 |
| Hispanic or Latino Population | | | | | | |
| 0-14 | 32 | 615,664 | 19 | 289,778 | 13 | 325,715 |
| 15-24 | 124 | 456,119 | 74 | 247,024 | 50 | 208,939 |
| 25-34 | 141 | 392,972 | 93 | 252,945 | 48 | 139,827 |
| 35-44 | 183 | 361,939 | 127 | 224,510 | 56 | 137,292 |
| 45-54 | 161 | 326,178 | 109 | 195,429 | 52 | 130,633 |
| 55-64 | 150 | 274,180 | 89 | 155,837 | 61 | 118,237 |
| 65+ | 168 | 333,135 | 86 | 190,376 | 82 | 142,610 |
| Total | 959 | 2760187 | 597 | 1555899 | 362 | 1203253 |

**Supplemental Table 7.** Optic Neuritis Cases and Baseline Populations in 2021

|  | All Sexes | | Females | | Males | |
| --- | --- | --- | --- | --- | --- | --- |
| Age | Optic Neuritis Cases | Baseline Population | Optic Neuritis Cases | Baseline Population | Optic Neuritis Cases | Baseline Population |
| All Races and Ethnicities | | | | | | |
| 0-14 | 293 | 4,352,667 | 150 | 2,020,797 | 143 | 2,261,214 |
| 15-24 | 875 | 3,901,112 | 523 | 2,083,938 | 342 | 1,718,090 |
| 25-34 | 1,597 | 4,191,932 | 1,075 | 2,487,396 | 480 | 1,575,204 |
| 35-44 | 2,242 | 4,327,198 | 1,522 | 2,488,622 | 660 | 1,701,297 |
| 45-54 | 2,504 | 4,124,095 | 1,635 | 2,300,830 | 767 | 1,686,396 |
| 55-64 | 2,633 | 4,858,771 | 1,632 | 2,607,928 | 919 | 2,074,815 |
| 65+ | 4,678 | 9,189,290 | 2,578 | 4,907,906 | 1,922 | 3,897,194 |
| Total | 14822 | 34945065 | 9115 | 18897417 | 5233 | 14914210 |
| White Population | | | | | | |
| 0-14 | 170 | 2,286,783 | 82 | 1,074,842 | 88 | 1,210,757 |
| 15-24 | 494 | 2,168,235 | 286 | 1,189,159 | 208 | 977,269 |
| 25-34 | 978 | 2,325,613 | 680 | 1,424,755 | 298 | 884,642 |
| 35-44 | 1,345 | 2,489,862 | 960 | 1,473,665 | 385 | 1,014,045 |
| 45-54 | 1,558 | 2,455,534 | 1,078 | 1,403,800 | 480 | 1,034,603 |
| 55-64 | 1697 | 3,093,510 | 1097 | 1,718,015 | 599 | 1,372,899 |
| 65+ | 3,251 | 6,380,562 | 1,858 | 3,510,927 | 1392 | 2,861,842 |
| Total | 9493 | 21200099 | 6041 | 11795163 | 3450 | 9356057 |
| Black Population | | | | | | |
| 0-14 | 55 | 762,577 | 29 | 361,562 | 26 | 400,637 |
| 15-24 | 154 | 625,708 | 104 | 347,866 | 50 | 277,322 |
| 25-34 | 249 | 680,051 | 175 | 428,357 | 74 | 251,243 |
| 35-44 | 401 | 667,062 | 274 | 409,599 | 127 | 257,049 |
| 45-54 | 397 | 617,733 | 270 | 366,987 | 127 | 250,400 |
| 55-64 | 435 | 694,767 | 280 | 396,411 | 155 | 297,985 |
| 65+ | 555 | 934,159 | 339 | 567,284 | 216 | 366,012 |
| Total | 2246 | 4982057 | 1471 | 2878066 | 775 | 2100648 |
| Hispanic or Latino Population | | | | | | |
| 0-14 | 52 | 754,042 | 20 | 356,437 | 32 | 397,389 |
| 15-24 | 148 | 566,320 | 95 | 308,749 | 53 | 257,360 |
| 25-34 | 187 | 484,508 | 133 | 303,375 | 54 | 180,908 |
| 35-44 | 206 | 450,898 | 140 | 271,078 | 66 | 179,661 |
| 45-54 | 206 | 407,264 | 131 | 238,086 | 75 | 169,046 |
| 55-64 | 153 | 329,109 | 88 | 185,378 | 65 | 143,633 |
| 65+ | 175 | 380,248 | 96 | 217,961 | 79 | 162,144 |
| Total | 1127 | 3372389 | 703 | 1881064 | 424 | 1490141 |

**Supplemental Table 8.** Optic Neuritis Cases and Baseline Populations in 2022

|  | All Sexes | | Females | | Males | |
| --- | --- | --- | --- | --- | --- | --- |
| Age | Optic Neuritis Cases | Baseline Population | Optic Neuritis Cases | Baseline Population | Optic Neuritis Cases | Baseline Population |
| All Races and Ethnicities | | | | | | |
| 0-14 | 391 | 5014645 | 203 | 2329191 | 187 | 2616903 |
| 15-24 | 1001 | 3733584 | 613 | 2043152 | 381 | 1605902 |
| 25-34 | 1775 | 3944925 | 1185 | 2418564 | 552 | 1416839 |
| 35-44 | 2572 | 4162462 | 1730 | 2471542 | 790 | 1572597 |
| 45-54 | 2745 | 4015202 | 1805 | 2295201 | 873 | 1599210 |
| 55-64 | 3046 | 4760481 | 1904 | 2590812 | 1072 | 2013667 |
| 65+ | 5259 | 9008044 | 2933 | 4855894 | 2173 | 3816389 |
| Total | 16789 | 34639343 | 10373 | 19004356 | 6028 | 14641507 |
| White Population | | | | | | |
| 0-14 | 228 | 2586421 | 123 | 1214361 | 104 | 1370972 |
| 15-24 | 572 | 2065470 | 347 | 1155889 | 225 | 907850 |
| 25-34 | 1087 | 2192497 | 723 | 1375438 | 364 | 815104 |
| 35-44 | 1559 | 2390614 | 1087 | 1448043 | 471 | 940762 |
| 45-54 | 1727 | 2390271 | 1190 | 1400150 | 537 | 988577 |
| 55-64 | 1994 | 3030738 | 1273 | 1695877 | 720 | 1332717 |
| 65+ | 3638 | 6239666 | 2089 | 3445651 | 1548 | 2787574 |
| Total | 10805 | 20895677 | 6832 | 11735409 | 3969 | 9143556 |
| Black Population | | | | | | |
| 0-14 | 66 | 861588 | 36 | 407889 | 30 | 453390 |
| 15-24 | 172 | 608538 | 119 | 346721 | 53 | 261370 |
| 25-34 | 284 | 648899 | 204 | 418658 | 80 | 229867 |
| 35-44 | 435 | 647063 | 297 | 407103 | 138 | 239654 |
| 45-54 | 435 | 603654 | 292 | 365439 | 143 | 237969 |
| 55-64 | 479 | 681837 | 318 | 394967 | 161 | 286552 |
| 65+ | 610 | 913081 | 354 | 559301 | 256 | 353047 |
| Total | 2481 | 4964660 | 1620 | 2900078 | 861 | 2061849 |
| Hispanic or Latino Population | | | | | | |
| 0-14 | 55 | 890779 | 27 | 419084 | 28 | 471524 |
| 15-24 | 170 | 538555 | 107 | 301324 | 63 | 237026 |
| 25-34 | 197 | 463453 | 133 | 301661 | 64 | 161593 |
| 35-44 | 251 | 424649 | 169 | 265764 | 82 | 158761 |
| 45-54 | 260 | 378832 | 166 | 229640 | 94 | 149085 |
| 55-64 | 181 | 312104 | 112 | 180377 | 69 | 131636 |
| 65+ | 229 | 370225 | 127 | 216496 | 102 | 153601 |
| Total | 1343 | 3378597 | 841 | 1914346 | 502 | 1463226 |

**Supplemental Table 9.** Optic Neuritis Cases and Baseline Populations in 2023

|  | All Sexes | | Females | | Males | |
| --- | --- | --- | --- | --- | --- | --- |
| Age | Optic Neuritis Cases | Baseline Population | Optic Neuritis Cases | Baseline Population | Optic Neuritis Cases | Baseline Population |
| All Races and Ethnicities | | | | | | |
| 0-14 | 484 | 4,806,253 | 257 | 2,227,344 | 226 | 2,492,106 |
| 15-24 | 967 | 3,146,441 | 572 | 1,743,789 | 384 | 1,305,392 |
| 25-34 | 1675 | 3,144,449 | 1073 | 1,921,662 | 531 | 1,098,673 |
| 35-44 | 2349 | 3,354,417 | 1561 | 1,969,787 | 701 | 1,250,108 |
| 45-54 | 2364 | 3,315,625 | 1642 | 1,878,548 | 765 | 1,300,624 |
| 55-64 | 2581 | 3,914,053 | 1539 | 2,106,104 | 932 | 1,633,129 |
| 65+ | 4450 | 7,150,754 | 2455 | 3,813,224 | 1803 | 2,975,826 |
| Total | 14870 | 28,831,992 | 9099 | 15,660,458 | 5342 | 12,055,858 |
| White Population | | | | | | |
| 0-14 | 267 | 2,477,196 | 140 | 1,167,194 | 127 | 1,309,502 |
| 15-24 | 518 | 1,740,754 | 314 | 996,555 | 204 | 743,264 |
| 25-34 | 1021 | 1,764,794 | 684 | 1,117,866 | 337 | 646,185 |
| 35-44 | 1454 | 1,952,815 | 1002 | 1,186,258 | 452 | 766,123 |
| 45-54 | 1472 | 2,002,773 | 989 | 1,178,438 | 483 | 824,143 |
| 55-64 | 1687 | 2,517,910 | 1060 | 1,411,888 | 627 | 1,105,780 |
| 65+ | 3118 | 4,982,339 | 1791 | 2,763,087 | 1327 | 2,218,583 |
| Total | 9537 | 17438581 | 5980 | 9821286 | 3557 | 7613580 |
| Black Population | | | | | | |
| 0-14 | 70 | 808,655 | 43 | 383,256 | 27 | 425,296 |
| 15-24 | 184 | 524,638 | 127 | 306,223 | 57 | 218,185 |
| 25-34 | 294 | 546,916 | 212 | 354,956 | 82 | 191,794 |
| 35-44 | 438 | 550,379 | 312 | 346,253 | 126 | 204,024 |
| 45-54 | 423 | 518,551 | 283 | 313,587 | 140 | 204,900 |
| 55-64 | 465 | 578,663 | 282 | 334,864 | 183 | 243,746 |
| 65+ | 596 | 747,093 | 349 | 458,888 | 247 | 288,106 |
| Total | 2470 | 4274895 | 1608 | 2498027 | 862 | 1,776,051 |
| Hispanic or Latino Population | | | | | | |
| 0-14 | 82 | 863,182 | 45 | 406,261 | 37 | 456,824 |
| 15-24 | 163 | 459,546 | 92 | 265,037 | 71 | 194,398 |
| 25-34 | 222 | 389,929 | 130 | 257,987 | 92 | 131,820 |
| 35-44 | 278 | 354,759 | 202 | 224,763 | 91 | 129,948 |
| 45-54 | 226 | 325,531 | 140 | 200,631 | 86 | 124,867 |
| 55-64 | 171 | 265,547 | 91 | 155,219 | 80 | 119,800 |
| 65+ | 216 | 306,503 | 119 | 179,883 | 97 | 126,588 |
| Total | 1358 | 2964997 | 819 | 1689781 | 554 | 1284245 |
